# Complete mitochondrial genome of the razor-backed musk turtle (*Sternotherus carinatus*, testudines: emydidae) in Korea

**DOI:** 10.1080/23802359.2023.2292744

**Published:** 2023-12-18

**Authors:** Jaehong Park, Seung-Ju Cheon, Jae-Hyuk Choi, Seung-Min Park, Ha-Cheol Sung, Dong-Hyun Lee

**Affiliations:** a School of Biological Sciences and Biotechnology, Chonnam National University, Gwangju, Korea; b Research Center of Ecomimetics, Chonnam National University, Gwangju, Korea; c Department of Biological Sciences, College of Natural Sciences, Chonnam National University, Gwangju, Korea

**Keywords:** *Sternotherus carinatus*, emydidae, mitochondrial genome

## Abstract

*Sternotherus carinatus* has been considered as a potential invasive species in Korea. However, the mitochondrial genome information of *S. carinatus* which can be used to control its effect on ecosystem is lacking. In this study, the complete mitochondrial genome of *S. carinatus* in Korea was sequenced and characterized. The mitochondrial genome consists of 37 genes (13 protein-coding genes, 22 transfer RNA genes, and 2 ribosomal RNA genes) and a noncoding region. Phylogenetic analysis based on the mitochondrial genome sequences showed that *S. carinatus* from Korea is separated from other turtles which are the invasive species in Korea. Sequence divergence calculations indicated near-zero divergence between *S. carinatus* populations in Korea, the USA, and China, suggesting limited genetic differentiation. In the context of the broader issue of invasive species disrupting ecosystems, this research contributes to the identification of mitochondrial genomes for various freshwater turtle species, emphasizing the need for extended data collection to discern genetic mixing trends between native and non-native species. This study is a significant step toward managing *S. carinatus* as a potential invasive species in Korea.

## Introduction

**Figure 1.**
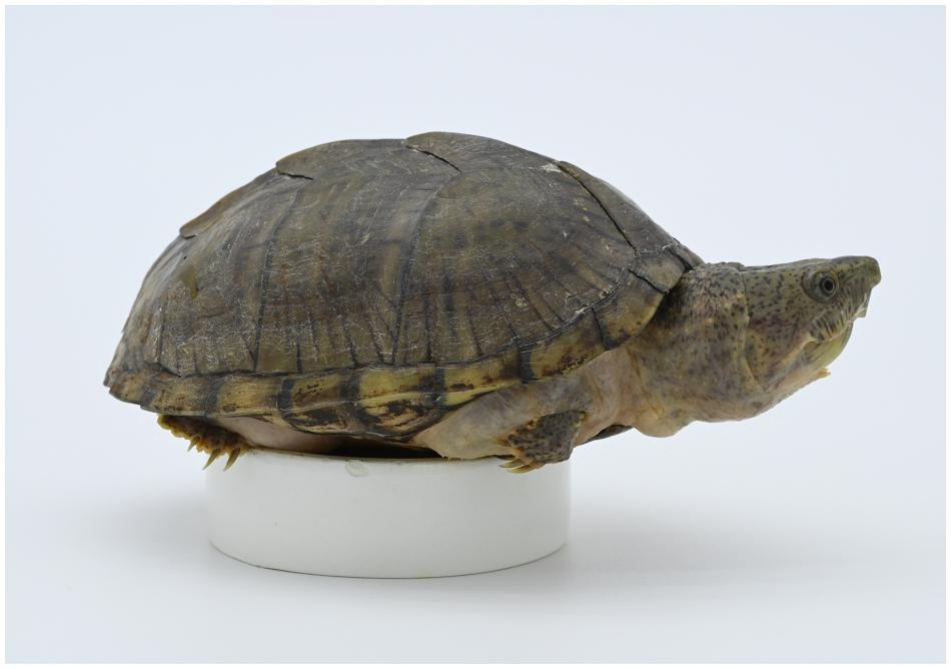
The image of *Sternotherus carinatus*. This picture was taken by Seung-Min Park.

The razor-backed musk turtle (*Sternotherus carinatus*, Gray 1856) is native to the USA (Lindeman 2008) (Figure 1). However, it has spread to other countries including Korea due to pet trade and intentional releases (Koo et al. 2020). This increase causes many problems in the native water ecosystem such as habitat and food competition, hybridization, and parasites (Cadi and Joly 2004; Silbernagel et al. 2013). Many researchers have tried to identify the mitochondrial genome of freshwater turtles and to decipher the relationships between native and non-native native species (Baek et al. 2022; Cheon et al. 2023; Chung et al. 2022; Kundu et al. 2022; Park et al. 2021, 2022; Ryu et al. 2021; Suzuki et al. 2014). Though not officially labeled as an invasive species in Korea, further discoveries of this species may lead to its designation as such. The comprehensive mitochondrial genome information of *S. carinatus* is crucial for effective species management and the protection of native species from potential impacts. Unfortunately, only limited information regarding the mitochondrial genome of *S. carinatus* is available. GenBank holds just one complete *S. carinatus* mitochondrial genome, alongside nine partial Cytb sequences, three partial 12S-rRNA sequences, two partial 16S-rRNA genes, one partial COX1 gene, and a complete tRNA-Val gene disclosed in GenBank.

In this study, we sequenced the complete mitochondrial genome of *S. carinatus*, providing valuable data for phylogenetic studies and exotic species management.

## Materials and methods

The *S. carinatus* specimen was captured from Gwangju (35°11′0.61″N, 126°53′26.64″E), Korea, and the tail tissue was acquired while the specimen was in a live state. This research received official sanction from Yeongsangang River Basin Environmental Office, a division of the Korea Ministry of Environment (permission number: 2021-8). Total genomic DNA was extracted from the tail tissue using the DNeasy Blood & Tissue kit (Qiagen, Valencia, CA) and deposited at the Museum of Wildlife, located in Research Center of Ecomimetics, Chonnam National University, Korea (https://biology.jnu.ac.kr; Ha-Cheol Sung; shcol2002@jnu.ac.kr) under species voucher number 2023-RCE-SC001. The mitochondrial genome was analyzed using the Illumina NovaSeq X plus platform (Illumina, San Diego, CA), which was performed by Macrogen (Seoul, Korea). The raw sequence data were assessed using FastQC and filtered to remove adaptor sequences, low-quality reads, reads with more than 10% unknown bases, and ambiguous bases using Trimmomatic v0.36 (Andrews 2010; Bolger et al. 2014). From 46,393,912 raw reads, 39,846,366 filtered reads were obtained. D*e novo* assembly was conducted with SPAdes 3.15.0, and the filtered reads were aligned using BLAST (Altschul et al. 1990; Bankevich et al. 2012). The coverage depth map is shown in Supplementary Figure 1, and the average read depth was 411.44. The complete sequence was annotated using the MITOS2 web server (Bernt et al. 2013).

To elucidate the relationship between *S. carinatus* and both native species and non-native species in Korea and investigate the phylogenetic position of *S. carinatus*, we selected *Chrysemys picta*, *Pseudemys peninsularis*, *P. concinna*, *Trachemys scripta*, *Mauremys sinensis*, and *S. carinatus* to represent the non-native species, while *M. reevesii* and *Pelodiscus sinensis* were chosen to represent the native species. We extracted their complete mitochondrial genome sequences from GenBank. All protein-coding genes (PCGs), rRNA genes, and tRNA genes were concatenated for analysis. The phylogenetic tree was constructed using MEGA X software (Kumar et al. 2018). Specifically, the sequences were aligned using the MUSCLE algorithm, and the phylogenetic tree was made using the maximum likelihood method and GTR + G model with 1000 bootstrap replicates (Edgar 2004; Waddell and Steel 1997). The GTR + G substitution model was selected as the best-fit model by MEGA X.

## Results

**Figure 2.**
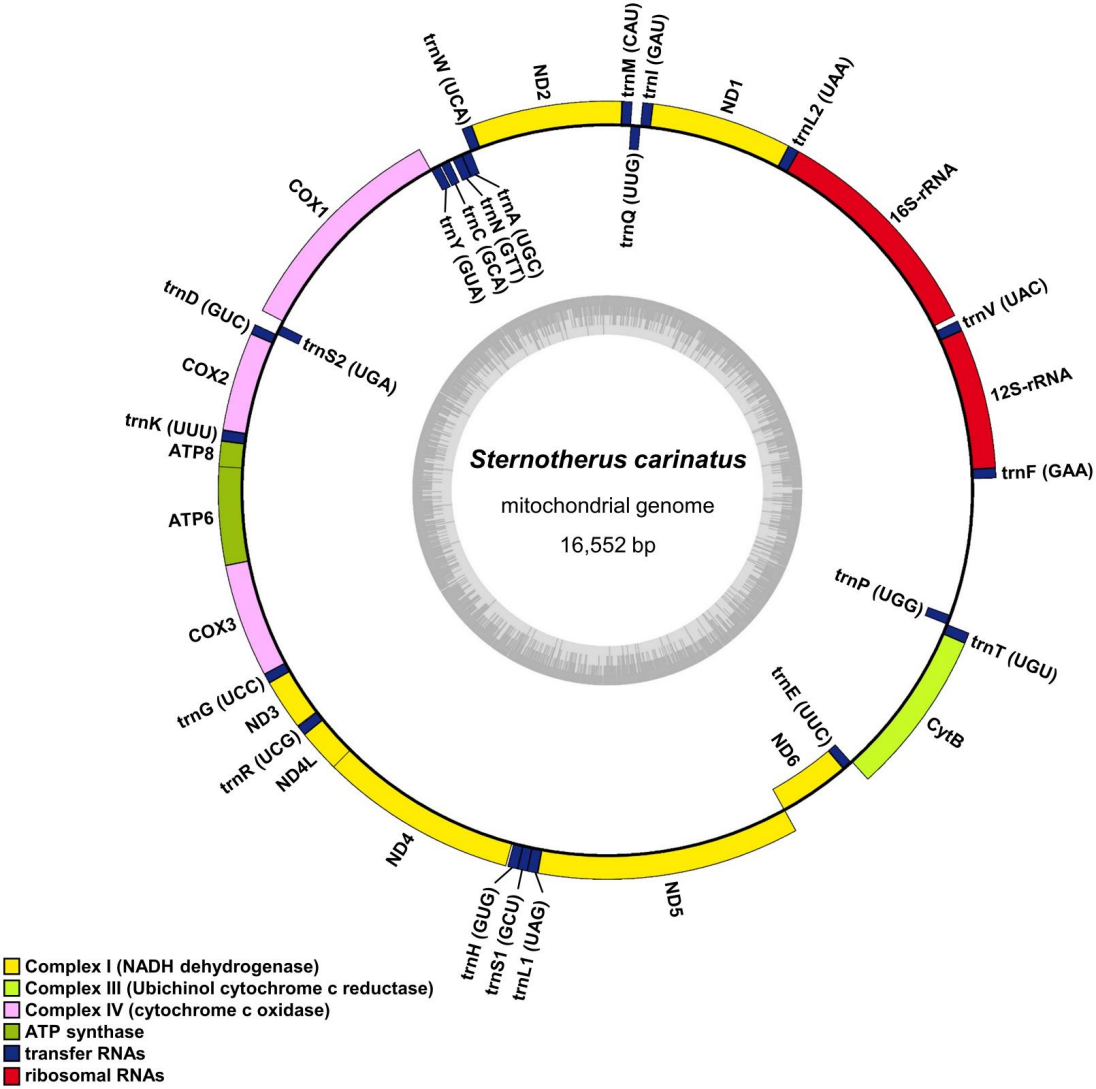
Mitochondrial genome map of *Sternotherus carinatus*. Genes encoded on the heavy strand are written outside the circle and genes on the light strand inside the circle. The dark and light parts in the grey circle represent GC and at contents, respectively.

The complete mitochondrial genome of *S. carinatus* spans 16,552 bp in length and deposited in GenBank (Accession number: OR253895). It comprises 13 PCGs, 22 transfer RNA (tRNA) genes, 2 ribosomal RNA (rRNA) genes, and a putative long non-coding control region. Notably, 12 PCGs, 14 tRNA genes, and 2 rRNA genes are encoded on the heavy strand, whereas 1 PCG (NADH dehydrogenase subunit 6) and 8 tRNA genes are encoded on the light strand (Figure 2). Most PCGs use canonical mitochondria start codons (AUG for ND2, COX1, COX2, ATP8, ATP6, COX3, ND3, ND5, ND6, and Cytb, and AUA for ND4L), except for ND1 and ND4, which have alternative initiation codon (AUU and GUG, respectively). Additionally, ND2, ND4, and COX1 have incomplete stop codons ending with U, and Cytb with UA.

The nucleotide composition of the *S. carinatus* mitochondrial genome is *A* = 35.8%, *T* = 27.3%, *G* = 12.0%, and *C* = 24.9%., which is identical to that of S. carinatus from China (HQ114563; *A* = 35.8%, *T* = 27.3%, *G* = 12.0%, and *C* = 24.9%) and similar to that of *Pelodiscus sinensis* from Korea (AY962573; *A* = 35.4%, *T* = 27.3%, *G* = 11.8%, and *C* = 25.6%). While *S. carinatus* from Korea shares a 99% sequence identity with the Chinese counterpart, it exhibits lower similarity with *Mauremys reevesii* from Korea (FJ469674; 78%), *Pseudemys concinna* from Korea (OM935747; 78%), or *Pelodiscus sinensis* from Korea (AY962573; 75%).

**Figure 3.**
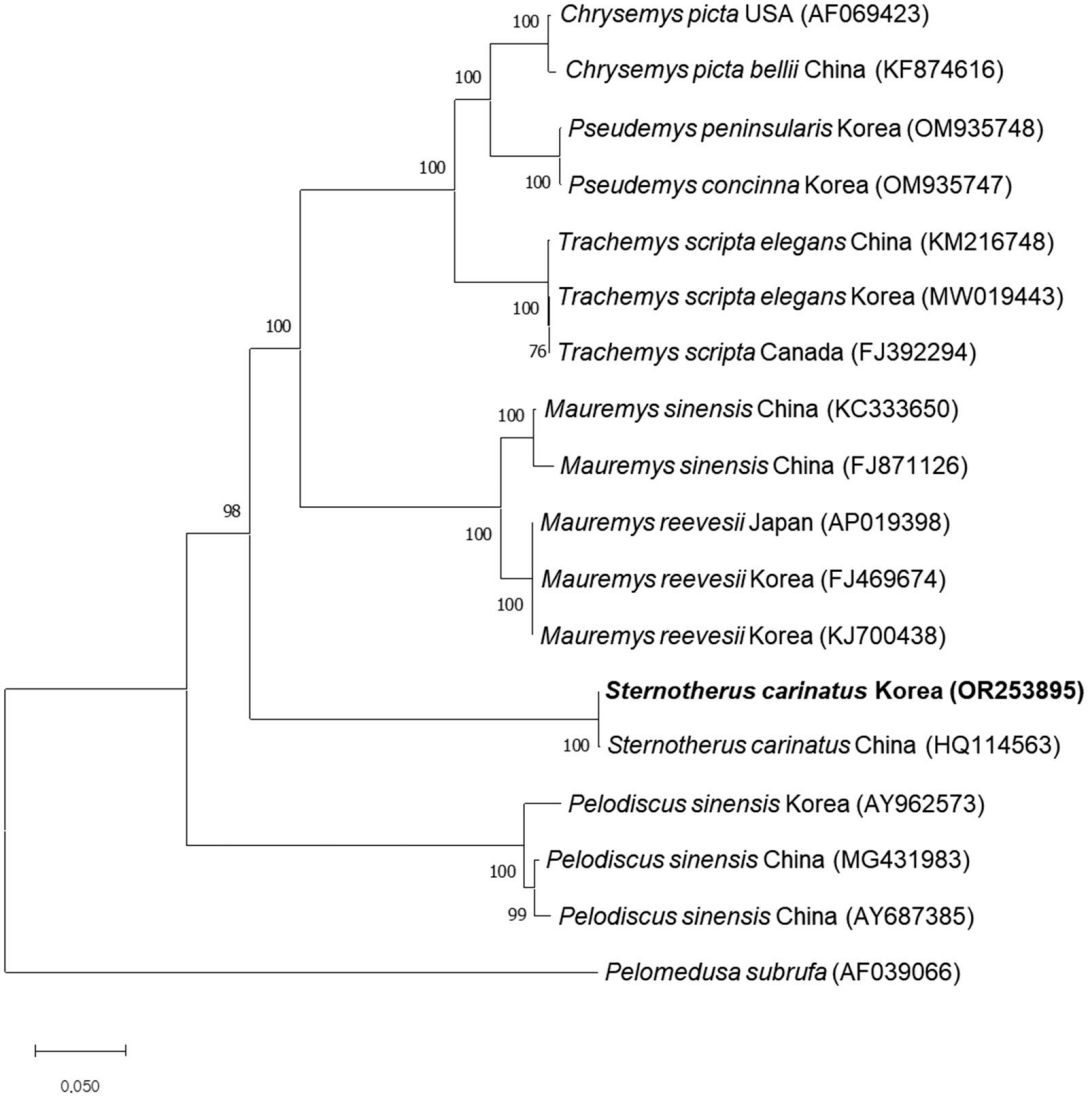
Phylogenetic tree of *S. carinatus* and other related species based on mitochondrial genome sequences. Phylogenetic analysis was performed using MEGA X software. The names of country referred behind each species indicate the locations where the sample was collected, as referenced on GenBank. GenBank accession numbers of each mt genome sequence are given in the bracket after the species name, and the following sequences were used: AF069423 (Mindell et al. 1999), KF874616 (Jiang et al. 2016), OM935748 (Chung et al. 2022), OM935747 (Park et al. 2022), KM216748 (Yu et al. 2016), MW019443 (Park et al. 2021), FJ392294 (Russell and Beckenbach 2008), KC333650 (Fang et al. 2013), FJ871126 (reference unavailable), AP019398 (Asami et al. 2019), FJ469674 (Shin et al. 2015), KJ700438 (reference unavailable), HQ114563 (Li et al. 2017), AY962573 (Jung et al. 2006), MG431983 (Zhang et al. 2019), AY687385 (reference unavailable), and AF039066 (Zardoya and Meyer 1998). the bootstrap value based on 1000 replicates is represented on each node. *Pelomedusa subrufa* was used as an outgroup to root the tree.

A phylogenetic tree, constructed using the complete mitochondrial genome sequence of *S. carinatus* from Korea along with sequences from 16 other species, corroborates the genetic similarity. *S. carinatus* from Korea clusters closely with *S. carinatus* from China while distinctly separating from other turtles, including *P. concinna*, *M. reevesii*, and *P. sinensis* (Figure 3).

We also determined the divergence between *S. carinatus* from Korea and China, yielding a divergence value of 0.067%. Furthermore, comparing Cytb sequences of *S. carinatus* available in GenBank indicates negligible or minimal divergence when comparing the Korean population with those from the USA (Table S1).

## Discussion and conclusion

We identified the complete mitochondrial genome of *S. carinatus* from Korea and studied its phylogenetic relationship with other turtles. In the phylogenetic tree, *S. carinatus* from Korea is placed in the distinct cluster apart from *M. reevesii* and *P. sinensis* which are the native species in Korea and *P. concinna* and *T. s. elegans* which are the invasive species in Korea. Furthermore, *S. carinatus* in Korea is separated from *S. carinatus* in China, despite being in the same cluster.

We calculated the sequence divergence between *S. carinatus* populations in Korea and those in the USA or China, revealing near-zero divergence. According to Morgan-Richards et al. (2017), a divergence exceeding 3% typically suggests significant population divergence over numerous generations. Considering the long lifespan of *S. carinatu*s (20–50 years) and the relatively recent occurrence of its trade, it appears that *S. carinatus* from non-native habitats like Korea and China may not exhibit substantial genetic differentiation from their native habitat counterparts. Nonetheless, accumulating mitochondrial genome data over an extended period is essential for a more comprehensive investigation into the impact of *S. carinatus* on native species.

The disturbance of ecosystems by invasive species is a severe issue in many countries including Korea. We and other scientific groups have culminated in the identification of mitochondrial genomes for various freshwater turtle species to understand their mitogenomic relationships. Typically, when comparing species from different genera, non-native species have been distinctly separated from the native species, indicating minimal genetic mixing from invasive species. Nonetheless, it is evident that more extensive and prolonged data collection is necessary. This study serves as a valuable contribution to the management of *S. carinatus*, a potential invasive species in Korea.

## Supplementary Material

Supplemental Material

Supplemental Material

## Data Availability

GenBank accession number from the complete mitochondrial genome of *Sternotherus carinatus* (OR253895) has been registered with the NCBI database (https://www.ncbi.nlm.nih.gov/OR253895). The associated BioProject, BioSample, and SRA accession numbers are PRJNA993827, SAMN36409325, and SRR25243167, respectively.

## References

[CIT0001] Altschul SF, Gish W, Miller W, Myers EW, Lipman DJ. 1990. Basic local alignment search tool. J Mol Biol. 215(3):403–410. doi: 10.1016/S0022-2836(05)80360-2. 2231712

[CIT0002] Andrews S. 2010. FastQC: a quality control tool for high throughput sequence data. Cambridge, UK: Babraham Bioinformatics, Babraham Institute.

[CIT0003] Asami M, Okuyama H, Takahashi J. 2019. Complete mitochondrial DNA sequence of the three-keeled pond turtle *Mauremys reevesii* (Reptilia: testudines). Mitochondrial DNA B. 4(1):1520–1521. doi: 10.1080/23802359.2019.1601511.

[CIT0004] Baek H. j, Kim P, Kim YC, Kim A, Kim S, Min MS, Lee H. 2022. The complete mitochondrial genome of the Amur soft-shelled turtle (*Pelodiscus maacki*i Brandt, 1858), from South Korea. Mitochondrial DNA B Resour. 7(3):498–500. doi: 10.1080/23802359.2022.2051759. 35311211 PMC8928817

[CIT0005] Bankevich A, Nurk S, Antipov D, Gurevich AA, Dvorkin M, Kulikov AS, Lesin VM, Nikolenko SI, Pham S, Prjibelski AD, et al. 2012. SPAdes: a new genome assembly algorithm and its applications to single-cell sequencing. J Comput Biol. 19(5):455–477. doi: 10.1089/cmb.2012.0021. 22506599 PMC3342519

[CIT0006] Bernt M, Donath A, Jühling F, Externbrink F, Florentz C, Fritzsch G, Pütz J, Middendorf M, Stadler PF. 2013. MITOS: improved de novo metazoan mitochondrial genome annotation. Mol Phylogenet Evol. 69(2):313–319. doi: 10.1016/j.ympev.2012.08.023. 22982435

[CIT0007] Bolger AM, Lohse M, Usadel B. 2014. Trimmomatic: a flexible trimmer for illumina sequence data. Bioinformatics. 30(15):2114–2120. doi: 10.1093/bioinformatics/btu170. 24695404 PMC4103590

[CIT0008] Cadi A, Joly P. 2004. Impact of the introduction of the red-eared slider (*Trachemys scripta elegans*) on survival rates of the European pond turtle (Emys orbicularis). Biodivers Conserv. 13(13):2511–2518. doi: 10.1023/B:BIOC.0000048451.07820.9c.

[CIT0009] Cheon SJ, Rahman MM, Lee JA, Park SM, Park JH, Lee DH, Sung HC. 2023. Confirmation of the local establishment of alien invasive turtle, *Pseudemys peninsularis*, in South Korea, using eggshell DNA. PLOS One. 18(2):e0281808. doi: 10.1371/journal.pone.0281808. 36795686 PMC9934327

[CIT0010] Chung D, Park J, Cheon S, Park SM, Sung HC, Lee DH. 2022. Complete mitochondrial genome of the peninsula cooter (*Pseudemys peninsularis*, Testudines: emydidae) in Korea. Mitochondrial DNA B Resour. 7(8):1441–1442. doi: 10.1080/23802359.2022.2107463. 35958058 PMC9359182

[CIT0011] Edgar RC. 2004. MUSCLE: multiple sequence alignment with high accuracy and high throughput. Nucleic Acids Res. 32(5):1792–1797. doi: 10.1093/nar/gkh340. 15034147 PMC390337

[CIT0012] Fang X, Tao B, Lin Y, Bao D, Zhang J. 2013. The complete mitochondrial genome of *Mauremys sinensis* (Testudines: geoemydidae). Mitochondrial DNA. 24(4):385–387. doi: 10.3109/19401736.2013.763239. 23379470

[CIT0013] Jiang JJ, Xia EH, Gao CW, Gao LZ. 2016. The complete mitochondrial genome of western painted turtle, *Chrysemys picta* bellii (Chrysemys, Emydidae). Mitochondrial DNA A DNA Mapp Seq Anal. 27(2):787–788. doi: 10.3109/19401736.2013.873900. 24438258

[CIT0014] Jung SO, Lee YM, Kartavtsev Y, Park IS, Kim DS, Lee JS. 2006. The complete mitochondrial genome of the Korean soft-shelled turtle *Pelodiscus sinensis* (Testudines, Trionychidae). DNA Seq. 17(6):471–483. doi: 10.1080/10425170600760091. 17381049

[CIT0015] Koo KS, Song S, Choi JH, Sung HC. 2020. Current distribution and status of non-native freshwater turtles in the wild, Republic of Korea. Sustainability. 12(10):4042. doi: 10.3390/su12104042.

[CIT0016] Kumar S, Stecher G, Li M, Knyaz C, Tamura K. 2018. MEGA X: molecular evolutionary genetics analysis across computing platforms. Mol Biol Evol. 35(6):1547–1549. doi: 10.1093/molbev/msy096. 29722887 PMC5967553

[CIT0017] Kundu S, Mukherjee T, Kim AR, Lee SR, Mukherjee A, Jung WK, Kim HW. 2022. Mitochondrial DNA and distribution modelling evidenced the lost genetic diversity and wild-residence of star tortoise, Geochelone elegans (Testudines: testudinidae) in India. Animals. 13(1):150. doi: 10.3390/ani13010150. 36611759 PMC9817980

[CIT0018] Li H, Liu J, Xiong L, Zhang H, Zhou H, Yin H, Jing W, Li J, Shi Q, Wang Y, et al. 2017. Phylogenetic relationships and divergence dates of softshell turtles (Testudines: trionychidae) inferred from complete mitochondrial genomes. J Evol Biol. 30(5):1011–1023. doi: 10.1111/jeb.13070. 28294452

[CIT0019] Lindeman PV. 2008. *Sternotherus carinatus* (Gray 1856)–Razorback musk turtle, razor-backed musk turtle. In: Rhodin AGJ, Pritchard PCH, Van Dijk PP, Saumure RA, Buhlmann KA, and Iverson JB (Eds). Conservation biology of freshwater turtles and tortoises: a compilation project of the IUCN/SSC tortoise and freshwater turtle specialist group. Chelonian research monographs. 012.1–012.6.

[CIT0020] Morgan-Richards M, Bulgarella M, Sivyer L, Dowle EJ, Hale M, McKean NE, Trewick SA. 2017. Explaining large mitochondrial sequence differences within a population sample. R Soc Open Sci. 4(11):170730. doi: 10.1098/rsos.170730. 29291063 PMC5717637

[CIT0021] Mindell DP, Sorenson MD, Dimcheff DE, Hasegawa M, Ast JC, Yuri T. 1999. Interordinal relationships of birds and other reptiles based on whole mitochondrial genomes. Syst Biol. 48(1):138–152. doi: 10.1080/106351599260490. 12078637

[CIT0022] Park J, Moon JI, Song YJ, Park SM, Cheon S, Sung HC, Lee DH. 2021. Complete mitochondrial genome of the red-eared slider (Trachemys scripta elegans, Testudines: emydidae) in Korea. Mitochondrial DNA B Resour. 6(3):918–919. doi: 10.1080/23802359.2021.1887773. 33796681 PMC7971311

[CIT0023] Park J, Cheon S, Park SM, Sung HC, Lee DH. 2022. Complete mitochondrial genome of the river cooter (Pseudemys concinna, Testudines: emydidae) in Korea. Mitochondrial DNA B Resour. 7(9):1659–1661. doi: 10.1080/23802359.2022.2119820. 36147372 PMC9487955

[CIT0024] Russell RD, Beckenbach AT. 2008. Recoding of translation in turtle mitochondrial genomes: programmed frameshift mutations and evidence of a modified genetic code. J Mol Evol. 67(6):682–695. doi: 10.1007/s00239-008-9179-0. 19030769 PMC2706983

[CIT0025] Ryu G, Moon JI, Song YJ, Park J, Park SM, Choi JH, Sung HC, Lee DH. 2021. Complete mitochondrial genome of the Cumberland slider (Trachemys scripta troostii, Testudienes: emydidae) in Korea. Mitochondrial DNA B Resour. 6(3):1131–1133. doi: 10.1080/23802359.2021.1902410. 33796765 PMC7995875

[CIT0026] Shin HW, Jang KH, Ryu SH, Choi EH, Hwang UW. 2015. Complete mitochondrial genome of the Korean reeves’s turtle *Mauremys reevesii* (Reptilia, Testudines, Geoemydidae). Mitochondrial DNA. 26(5):676–677. doi: 10.3109/19401736.2013.840603. 24102604

[CIT0027] Silbernagel C, Clifford DL, Bettaso J, Worth S, Foley J. 2013. Prevalence of selected pathogens in western pond turtles and sympatric introduced red-eared sliders in California, USA. Dis Aquat Organ. 107(1):37–47. doi: 10.3354/dao02663. 24270022

[CIT0028] Suzuki D, Yabe T, Hikida T. 2014. Hybridization between *Mauremys japonica* and *Mauremys reevesii* inferred by nuclear and mitochondrial DNA analyses. J Herpetol. 48(4):445–454. doi: 10.1670/11-320.

[CIT0029] Waddell PJ, Steel MA. 1997. General time-reversible distances with unequal rates across sites: mixing gamma and inverse Gaussian distributions with invariant sites. Mol Phylogenet Evol. 8(3):398–414. doi: 10.1006/mpev.1997.0452. 9417897

[CIT0030] Yu D, Fang X, Storey KB, Zhang Y, Zhang J. 2016. Complete mitochondrial genomes of the yellow-bellied slider turtle *Trachemys scripta* and anoxia tolerant red-eared slider *Trachemys scripta* elegans. Mitochondrial DNA Part A. 27(3):2276–2277. 10.3109/19401736.2014.984178 25541313

[CIT0031] Zhang J, Zhou Q, Yang X, Yu P, Zhou W, Gui Y, Ouyang X, Wan Q. 2019. Characterization of the complete mitochondrial genome and phylogenetic analysis of *Pelodiscus sinensis*, a mutant Chinese soft-shell turtle. Conservation Genet Resour. 11(3):279–282. doi: 10.1007/s12686-018-1007-2.

[CIT0032] Zardoya R, Meyer A. 1998. Complete mitochondrial genome suggests diapsid affinities of turtles. Proc Natl Acad Sci U S A. 95(24):14226–14231. doi: 10.1073/pnas.95.24.14226. 9826682 PMC24355

